# Development of a multimodal system based on acoustic analysis and EEG signals for early detection of Alzheimer's disease: integrated study of acoustic and neuronal biomarkers

**DOI:** 10.1002/alz70856_102116

**Published:** 2025-12-24

**Authors:** Nayrouz Denguir, Amina Bouraoui

**Affiliations:** ^1^ Higher Institute of Medical Technologies of Tunis, tunis, tunisia, Tunisia; ^2^ Riadi, GLORY, National School of Computer Sciences, ENSI University of Manouba 1 Institut Supérieur des Technologies Médicales‐ISTMT University of Tunis El Manar 9 rue Zouhair Essafi, Tunis, Tunisia 2, tunis, tunisia, Tunisia

## Abstract

**Background:**

Late‐stage diagnosis of Alzheimer's disease (AD) limits the effectiveness of current treatments. Traditional diagnostic methods (e.g., cognitive tests, neuroimaging) are invasive and often detect AD too late for effective early intervention. Recent research highlights the potential of non‐invasive biomarkers, such as acoustic signals and EEG, for earlier detection. Studies [1] show that subtle speech changes (e.g., pitch instability, altered pauses) can indicate early cognitive decline, while studies [2, 3, 4] demonstrate that EEG can reliably detect early neuronal anomalies. This study proposes a multimodal system integrating acoustic and EEG biomarkers to improve diagnostic accuracy and enable earlier interventions.

**Method:**

The proposed system operates in four phases: Collection of vocal recordings and EEG signals from both Alzheimer's disease patients and healthy controls. Preprocessing, involving the extraction of acoustic features (e.g., pitch and stability) and neuronal patterns from the EEG signals. Multimodal data fusion, where these extracted features are integrated using machine learning techniques to improve detection accuracy. Validation through cross‐validation and performance metrics to ensure system robustness and reliability.

**Result:**

Preliminary analyses indicate that combining acoustic and EEG biomarkers can significantly improve the sensitivity of AD detection compared to using either modality alone. By detecting subtle cognitive and neuronal changes at earlier stages, this system has the potential to assist clinicians in more effective AD diagnosis. Its non‐invasive design makes it suitable for clinical use, enabling improved patient management and intervention strategies.

**Conclusion:**

Integrating acoustic and EEG biomarkers presents a novel approach to non‐invasive, early‐stage Alzheimer's detection. By utilizing complementary data from speech and brain activity, this multimodal system offers valuable insights for clinicians, facilitating timely interventions and improved patient outcomes.

**References**

1. NIA, Subtle changes in speech are associated with early signs of Alzheimer's disease in the brain, NIH, 2024

2. Xiaobo Mao, Diagnosis of Alzheimer's disease via resting‐state EEG: integration of spectrum, complexity, and synchronization signal features, Frontiers, 2023

3. Yang DW, Ho S, Hong YJ, Jeong JH, Park KH, Kim S, Wang MJ, Choi SH, Kang SW, ShimY Electroencephalography for Early Detection of Alzheimer's Disease in Subjective Cognitive Decline, Korean Dementia Association, 2022Oct25;21(4):126–137

4. Luke Tait, Francesco Tamagnini, George Stothart, Edoardo Barvas, Chiara Monaldini, Roberto Frusciante, Mirco Volpini, Susanna Guttmann, Elizabeth Coulthard, Jon T. Brown, Nina Kazanina & Marc Goodfellow, EEG microstate complexity for aiding early diagnosis of Alzheimer's disease, scientific reports, 2020

